# Supplementary material for: An open-source framework for large-scale, flexible evaluation of biomedical text mining systems
Source: J Biomed Discov Collab. 2008 Jan 29;3:1. doi: 10.1186/1747-5333-3-1 (PMC2276192; doi:10.1186/1747-5333-3-1)
Supplement: Additional file 1 — Evaluation platform source code. The additional file contains the software evaluation framework discussed in this paper. The most current release can be downloaded from SourceForge.net [1]. See the accompanying README file for instructions on installation and use. Also included in the distribution is a collection of UIMA wrappers for some commonly used BioNLP tools and annotated corpora. [file 1747-5333-3-1-S1.zip › bionlp_uima_repository-1.0.1/docs/api/src-html/edu/uchsc/ccp/util/nlp/ei/xml/XMLEntityExtractor.html]

```
001    package edu.uchsc.ccp.util.nlp.ei.xml;
002    
003    /*
004     * This class is utilized by KeX gene tagger and PennBioTaggers
005     */
006    
007    import java.util.ArrayList;
008    import java.util.List;
009    import java.util.regex.Matcher;
010    import java.util.regex.Pattern;
011    
012    import edu.uchsc.ccp.util.nlp.GenericTextUtils;
013    import edu.uchsc.ccp.util.nlp.annotation.AnnotationSet;
014    import edu.uchsc.ccp.util.nlp.annotation.Annotator;
015    import edu.uchsc.ccp.util.nlp.annotation.TextAnnotation;
016    import edu.uchsc.ccp.util.nlp.mention.ClassMention;
017    
018    public class XMLEntityExtractor {
019        private static final boolean DEBUG = false;
020    
021        public static List<TextAnnotation> parseEntitiesFromSparseXML(String taggedText, String originalText, String tag, String documentID,
022                String classMentionType, Annotator annotator) {
023            List<TextAnnotation> returnHits = new ArrayList<TextAnnotation>();
024            try {
025                /* Initialize the Regex that will be used for finding the text thought to be protein names */
026                // Pattern entityPattern = Pattern.compile("<" + tag + ">(.*?)</" + tag + ">");
027                Pattern entityPattern = Pattern.compile("<" + tag + ">([^(</)]+)</" + tag + ">");
028                Matcher entityMatcher;
029    
030                /* Find all of the tagged protein names by searching for <p>protein names inside</p> in the KeX output */
031                entityMatcher = entityPattern.matcher(taggedText);
032                String entityText = "";
033    
034                /*
035                 * In order to ensure we capture duplicate mentions of the same protein name in the text, we must keep track of where we start
036                 * to search for matches in the text. If returnHits is empty, then start looking from zero, else start looking from the end
037                 * index of the last annotation added to the returnHits list
038                 */
039                int startLookingForMatchesAtIndex = 0;
040    
041                while (entityMatcher.find()) {
042                    entityText = entityMatcher.group(1);
043                    if (DEBUG) {
044                        System.out.println("Found a protein... text= " + entityText + " start_index= " + entityMatcher.start());
045                    }
046    
047                    /* Find all instances of the found protein text within the original text */
048                    List<TextAnnotation> entityAnnotations = GenericTextUtils.findTargetInText(entityText, originalText,
049                            startLookingForMatchesAtIndex, documentID, classMentionType, annotator, false);
050    
051                    /* and add them to the list to return. */
052                    returnHits.add(entityAnnotations.get(0));
053    
054                    if (returnHits.size() == 0) {
055                        System.out.println("***********   missed entity: " + entityText + "  ***********");
056                    }
057    
058                    /* Update startLookingForMatchesAtIndex to be the end of the span of the most recent additiong to the returnHits lise. */
059                    if (returnHits.size() > 0) {
060                        startLookingForMatchesAtIndex = returnHits.get(returnHits.size() - 1).getAnnotationSpanEnd();
061                    }
062                }
063    
064            } catch (Exception e) {
065                System.err.println("Error while parsing KeX output file. KeXEISystem.");
066                e.printStackTrace();
067            }
068            return returnHits;
069    
070        }
071    
072        /**
073         * Use when trying to get entity spans from xml that has not been tokenized (you therefore do not need the original text)
074         * NOTE: Due to the primitive nature of this code, be sure to remove all xml tags except for the one you are looking for.
075         * @param taggedText
076         * @param openTagText
077         * @param closeTagText
078         * @param documentID
079         * @param documentCollectionID
080         * @param classMentionType
081         * @param annotator
082         * @param annotationSet
083         * @return
084         */
085        public static List<TextAnnotation> parseEntitiesFromSparseUntokenizedXML(String taggedText, String openTagText, String closeTagText,
086                String documentID, int documentCollectionID, String classMentionType, Annotator annotator, AnnotationSet annotationSet) {
087            return parseEntitiesFromSparseUntokenizedXML(taggedText, openTagText, closeTagText, documentID, documentCollectionID, classMentionType,annotator, annotationSet, false);
088        }
089        
090        public static List<TextAnnotation> parseEntitiesFromSparseUntokenizedXML(String taggedText, String openTagText, String closeTagText,
091                String documentID, int documentCollectionID, String classMentionType, Annotator annotator, AnnotationSet annotationSet, boolean removeInternalXMLTags) {
092            List<TextAnnotation> entityList = new ArrayList<TextAnnotation>();
093    
094            int offset = 0;
095    
096            int startTagLength = openTagText.length() + 2; /* +2 for the < and > */
097            int endTagLength = closeTagText.length() + 3; /* +2 for the < and >, +1 for the / */
098    
099            
100            openTagText = GenericTextUtils.escapeCharacterForRegEx(openTagText);
101            closeTagText = GenericTextUtils.escapeCharacterForRegEx(closeTagText);
102            
103            Pattern p = Pattern.compile("<" + openTagText + ">(.*?)</" + closeTagText + ">");
104            Matcher m = p.matcher(taggedText);
105    
106            while (m.find()) {
107                int matchStart = m.start(1);
108                int matchEnd = m.end(1);
109    
110                String entityStr = m.group(1);
111                
112                int internalStartOffset = 0;
113                int internalEndOffset = 0;
114                
115                /* we may want to remove any remaining xml tags if they are present */
116                if (removeInternalXMLTags) {
117                    Pattern xmlTagPattern = Pattern.compile("<(.*?)>(.*?)</(.*?)>");
118                    Matcher xmlTagMatcher = xmlTagPattern.matcher(entityStr);
119                    List<String> tagsToRemove = new ArrayList<String>();
120                    int firstTagStartIndex = 999999999;
121                    int firstTagLength = -1;
122                    while (xmlTagMatcher.find()) {
123                        String startTag = xmlTagMatcher.group(1);
124                        if (xmlTagMatcher.start(1) < firstTagStartIndex) {
125                            firstTagStartIndex = xmlTagMatcher.start(1)-1;
126                            firstTagLength = startTag.length() + 2;  /* + 2 for the < and > */
127                        }
128                        String endTag = xmlTagMatcher.group(3);
129                        /* make sure the tags match - this is a safety check for removal */
130                        if (startTag.equals(endTag)) {
131                            tagsToRemove.add(startTag);
132                        }
133                    }
134                    
135                    /* remove internal xml tags that were found */
136                    int offsetAdjustment = 0;
137                    for (String tagStr : tagsToRemove) {
138                        entityStr = entityStr.replaceAll("<" + tagStr + ">", "");
139                        entityStr = entityStr.replaceAll("</" + tagStr + ">", "");
140                        offsetAdjustment += (tagStr.length()*2 + 5);
141                    }
142                    
143                    /* now adjust span to account for xml tag removal */
144                    if (firstTagStartIndex == 0) {
145                        /* there was an xml tag at the beginning of the entity, so we must adjust the start index */
146                        internalStartOffset = firstTagLength;
147                    }
148                    
149                    internalEndOffset = offsetAdjustment;
150                    
151                }
152                
153                int entityStart = matchStart - startTagLength - offset - internalStartOffset;
154                int entityEnd = matchEnd - startTagLength - offset - internalEndOffset;
155    
156                
157                TextAnnotation ta = createAnnotation(documentID, documentCollectionID, entityStart, entityEnd, entityStr, classMentionType,
158                        annotator, annotationSet);
159    
160                entityList.add(ta);
161    
162                /* adjust offset */
163                offset += (startTagLength + endTagLength);
164            }
165    
166            return entityList;
167        }
168    
169        private static TextAnnotation createAnnotation(String documentID, int documentCollectionID, int spanStart, int spanEnd,
170                String coveredText, String classMentionType, Annotator annotator, AnnotationSet annotationSet) {
171            TextAnnotation ta = new TextAnnotation();
172    
173            /* set the annotator */
174            ta.setAnnotator(annotator);
175    
176            /* add the annotation set */
177            ta.addAnnotationSet(annotationSet);
178    
179            /* set the documentID */
180            ta.setDocumentID(documentID);
181    
182            /* set the documentCollectionID */
183            ta.setDocumentCollectionID(documentCollectionID);
184    
185            /* set the span */
186                ta.setAnnotationSpanEnd(spanEnd);
187                ta.setAnnotationSpanStart(spanStart);
188    
189            /* set the covered text */
190            ta.setCoveredText(coveredText);
191    
192            /* set the class mention */
193            ClassMention cm = new ClassMention(classMentionType);
194            ta.setClassMention(cm);
195    
196            return ta;
197        }
198    
199    }
```
